# Reinforcement Learning on Slow Features of High-Dimensional Input Streams

**DOI:** 10.1371/journal.pcbi.1000894

**Published:** 2010-08-19

**Authors:** Robert Legenstein, Niko Wilbert, Laurenz Wiskott

**Affiliations:** 1 Institute for Theoretical Computer Science, Graz University of Technology, Graz, Austria; 2 Institute for Theoretical Biology, Humboldt-Universität zu Berlin, Berlin, Germany; 3 Bernstein Center for Computational Neuroscience, Berlin, Germany; 4 Institut für Neuroinformatik, Ruhr-Universität Bochum, Bochum, Germany; RIKEN Brain Science Institute, Japan

## Abstract

Humans and animals are able to learn complex behaviors based on a massive stream of sensory information from different modalities. Early animal studies have identified learning mechanisms that are based on reward and punishment such that animals tend to avoid actions that lead to punishment whereas rewarded actions are reinforced. However, most algorithms for reward-based learning are only applicable if the dimensionality of the state-space is sufficiently small or its structure is sufficiently simple. Therefore, the question arises how the problem of learning on high-dimensional data is solved in the brain. In this article, we propose a biologically plausible generic two-stage learning system that can directly be applied to raw high-dimensional input streams. The system is composed of a hierarchical slow feature analysis (SFA) network for preprocessing and a simple neural network on top that is trained based on rewards. We demonstrate by computer simulations that this generic architecture is able to learn quite demanding reinforcement learning tasks on high-dimensional visual input streams in a time that is comparable to the time needed when an explicit highly informative low-dimensional state-space representation is given instead of the high-dimensional visual input. The learning speed of the proposed architecture in a task similar to the Morris water maze task is comparable to that found in experimental studies with rats. This study thus supports the hypothesis that slowness learning is one important unsupervised learning principle utilized in the brain to form efficient state representations for behavioral learning.

## Author Summary

Humans and animals are able to learn complex behaviors based on a massive stream of sensory information from different modalities. Early animal studies have identified learning mechanisms that are based on reward and punishment such that animals tend to avoid actions that lead to punishment whereas rewarded actions are reinforced. It is an open question how sensory information is processed by the brain in order to learn and perform rewarding behaviors. In this article, we propose a learning system that combines the autonomous extraction of important information from the sensory input with reward-based learning. The extraction of salient information is learned by exploiting the temporal continuity of real-world stimuli. A subsequent neural circuit then learns rewarding behaviors based on this representation of the sensory input. We demonstrate in two control tasks that this system is capable of learning complex behaviors on raw visual input.

## Introduction

The nervous system of vertebrates continuously generates decisions based on a massive stream of complex multimodal sensory input. The strength of this system is based on its ability to adapt and learn suitable decisions in novel situations. Early animal studies have identified learning mechanisms that are based on reward and punishment such that animals tend to avoid actions that lead to punishment whereas rewarded actions are reinforced. The study of such reward-based learning goes back to Thorndikes law of effect [1]. Later, the mathematically well-founded theory of reinforcement learning, which describes learning by reward, has been developed [2], [3].

In a general reinforcement learning problem, an agent senses the environment at time 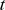 via a state 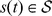, where 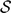 is the state space of the problem. The agent then chooses an action 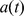, which leads to state 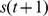 according to some (in general probabilistic) state-transition relation. The agent also receives some reward signal 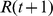, which depends probabilistically on the state 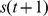. By choosing an action 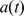 the agent aims at maximizing the expected discounted future reward
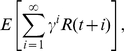
where 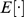 denotes the expectation and 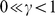 is some discount rate. This general theory has a huge influence on psychology, systems neuroscience, machine learning, and engineering and numerous algorithms have been developed for the reinforcement learning problem. By utilizing these algorithms, many impressive control applications have been developed. Several experimental studies connect the neural basis for reward-based learning in animals to well-known reinforcement learning algorithms. It has been shown that the activity of dopaminergic neurons in the ventral tegmental area is related to the reward-prediction error [4], a signal that is needed for parameter updates in temporal difference learning [3]. These neurons in turn have dense diffuse projections to several important areas including the striatum. In the striatum it was shown that dopamine influences synaptic plasticity [5]. Hence, the principal basis of reward-based learning in this sub-system, although not well understood yet, could be related to well-known reinforcement learning algorithms. However, the learning capabilities of animals such as rodents are still far from reach with current reinforcement learning algorithms. Since physiological experiments are consistent with quite standard reward-based learning schemes, it is reasonable to speculate that the superior learning capabilities of animals is to a high degree based on the ability to autonomously extract relevant features from the input stream such that subsequent reward-based learning is highly simplified (We note that the distinction between feature extraction and reward-based learning is most likely not so strict in the brain. For example, acetylcholine is a prominent neuromodulator in sensory cortical areas which could be utilized for task-dependent feature extraction). In fact, one of the most crucial design questions in the design of a reinforcement learning system is the definition of the state space 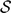. Most reinforcement learning algorithms are only applicable if the state space of the problem is sufficiently small. Thus, if the sensory input to a controller is complex and high-dimensional, the first task of the designer is to extract from this high-dimensional input stream a highly compressed representation that encodes the current state of the environment in a suitable way such that the agent can learn to solve the task. In contrast, the nervous system is able to learn good decisions from high-dimensional visual, auditory, tactile, olfactory, and other sensory inputs autonomously. The autonomous extraction of relevant features in the nervous system is commonly attributed to neocortex. The way how neocortex extracts features from the sensory input is still unknown and a matter of debate. Several principles with biologically plausible neural implementations have been postulated. Possible candidates are for example principal component analysis (PCA) [6], [7], independent component analysis [8]–[10], and information bottleneck optimization [10], [11]. One learning algorithm that exploits slowness information is slow feature analysis (SFA) [12]. SFA extracts the most slowly varying features in the input stream (see below). One important property of SFA is that it can be applied in a hierarchical fashion, first extracting local features on the raw input data which are then integrated to more and more global and abstract features. This hierarchical organization is similar to cortical organization for example in the visual system (we note however that the characteristic recurrent organization of cortex where multiple loops provide feedback from higher-level to lower-level processing is not yet exploited in hierarchical SFA architectures). Furthermore, the features that emerge from SFA have been shown to resemble the stimulus tunings of neurons both at low and high levels of sensory representation such as various types of complex cells in the visual system [13] as well as hippocampal place cells, head-direction cells, and spatial-view cells [14].

These features have been extracted from visual input. This hints at the usefulness of SFA for autonomous learning on high-dimensional input streams. In fact, it was shown in [15] that important stimulus features such as object category, the position of objects, or their orientation can be easily extracted by supervised training with high precision from the slow features of a high-dimensional visual input stream. It should be noted that the SFA algorithm is only one particular implementation of learning based on slowness, and there have been various earlier approaches, e.g., [16]–[19]. Slowness has previously been used in some hierarchical models as well [20]–[22].

Unsupervised learning based on the slowness principle (i.e., learning that exploits temporal continuity of real-world stimuli) has recently attracted the attention of experimentalists [23], [24]. It was shown in monkey experiments, that features in monkey infero temporal cortex are adapted in a way that is consistent with the slowness principle [23].

In this article, we propose a learning system where the state space representation is constituted autonomously by SFA. A subsequent neural circuit is then trained by a reward-based synaptic learning rule that is related to policy gradient methods or Q-learning in classical reinforcement learning. We apply this system to two closed-loop control tasks where the input to the system is high-dimensional raw pixel data and the output are motor commands. We thus show in this article for two control tasks on high-dimensional visual input streams that the representation of the SFA output is well suited to serve as a state-representation for reward-based learning in a subsequent neural circuit.

## Methods

**Figure 1:**
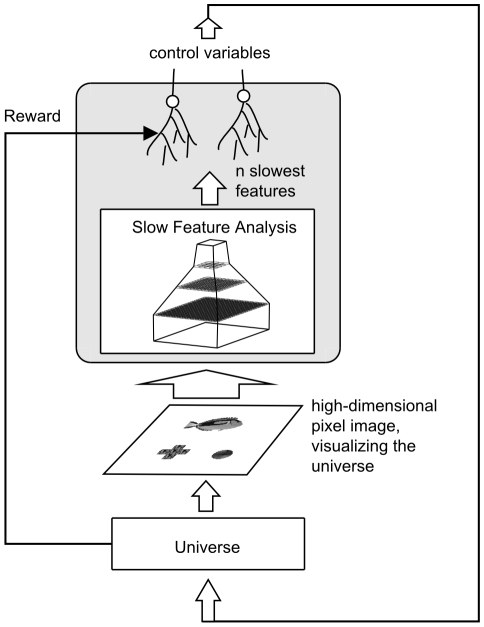
The learning system and the simulation setup. The learning system (gray box) consists of a hierarchical slow-feature analysis network, which reduces the dimensionality of the high-dimensional visual input. This reduction is trained in an unsupervised manner. The extracted features from the SFA network serve as inputs for a small neural network that produces the control commands. This network is trained by simple reward-modulated learning. We tested the learning system in a closed-loop setup. The system controlled an agent in an environment (universe). The state of the environment was accessible to the learning system via a visual sensory stream of dimension 155
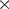
155. A reward signal was made accessible to the control network for learning.

The learning system considered in this article consists of two components, a hierarchical SFA network and a subsequent control network, see Figure 1. The SFA network reduces the dimensionality of the state-space from 24025 to a small number 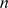 that was chosen to be 64 or less in this article. The decisions of the subsequent control network are based solely on the features extracted by the SFA network.

### The environment

**Figure 2:**
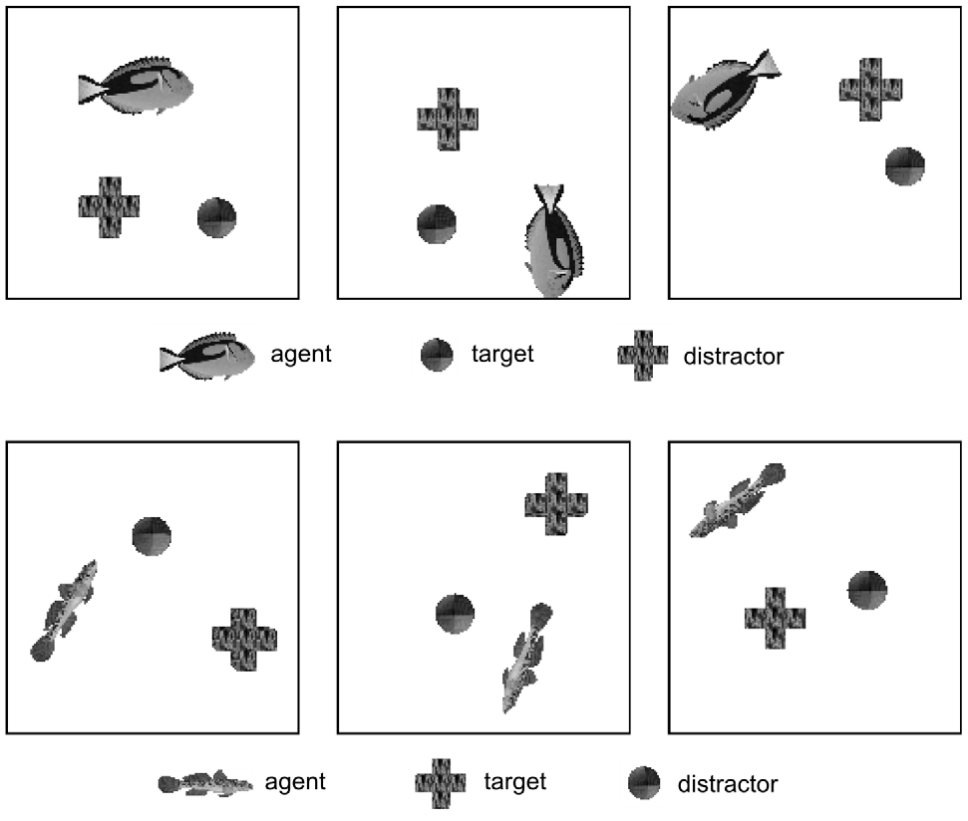
Examples for the visual input to the learning system for the variable-targets task. The scene consists of three objects, the agent (fish), an object that indicates the location of the target, and a second object that acts as a distractor. As indicated in the figure the target object depends on the fish identity. For the fish identity shown in the upper panels the target is always the disk, whereas the for the other fish identity, the target is the cross. In the visual input for the water-maze task the target and the distractor are not present, and the agent representation is the non-rotated image of the fish-type shown in the upper panels.

We tested this learning system on two different control tasks where an agent (a fish) navigates in a 2D environment with analog state- and action-space: a task similar to the Morris water-maze task [25] and a variable-targets task, see section “Tasks”. The state of the universe at time 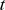 (see below for details) was used to render a 155 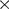 155 dimensional 2D visual scene that showed the agent (a fish; for one of the tasks two fish-types with different visual appearance were used) at a position 
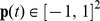
 and potentially other objects, see Figure 2. This visual scene constituted the input to the learning system. These tasks are to be seen as generic control tasks of reasonable complexity. The bird's eye perspective used here is of course not realistic for animal agents. As demonstrated in [14] our model should also be able to deal with a first-person perspective, especially in the Morris water-maze. For the variable-targets task this would introduce some complications like the target not being in the field of view or being hidden behind the distractor. On the other hand it would simplify the task, since the agent would not need to know its own position and angle (it could simply center its field of view on the target).

For the training of the system, we distinguish two different phases. In a first phase the SFA network is trained. In this phase, the fish, the target, and the distractor are floating slowly over the 2D space of the environment. The type of fish is changed from time to time (see section “Training stimuli of the hierarchical network”).

In a second phase the control circuit is trained. This phase consists of several learning episodes, an episode being one trial to reach a defined target from the initial fish-position. An episode ends when the target is reached or when a maximum number of 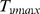 time-steps is exceeded.

### Slow feature analysis

The hierarchical network described in the next section is based on the Slow Feature Analysis Algorithm (SFA) [26], [27]. SFA solves the following learning task: Given a multidimensional input signal we want to find instantaneous scalar input-output functions that generate output signals that vary as slowly as possible but still carry significant information. To ensure the latter we require the output signals to be uncorrelated and have unit variance. In mathematical terms, this can be stated as follows:

**Optimization problem:** *Given a function space* 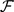 *and an I-dimensional input signal* 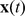 *find a set of* 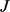 *real-valued input-output functions* 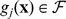 *such that the output signals* 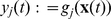


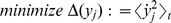
(1)
*under the constraints*

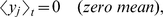
(2)

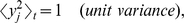
(3)

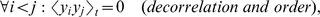
(4)
*with* 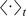 *and* 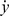 *indicating temporal averaging and the derivative of* 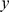, *respectively.*

Equation (1) introduces the 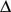-value, which is a measure of the temporal slowness (or rather fastness) of the signal 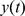. It is given by the mean square of the signal's temporal derivative, so that small 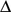-values indicate slowly varying signals. The constraints (2) and (3) avoid the trivial constant solution and constraint (4) ensures that different functions 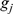 code for different aspects of the input. Because of constraint (4) the 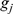 are also ordered according to their slowness, with 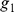 having the smallest 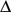.

It is important to note that although the objective is slowness, the functions 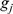 are instantaneous functions of the input, so that slowness cannot be achieved by low-pass filtering. Slow output signals can only be obtained if the input signal contains slowly varying features that can be extracted instantaneously by the functions 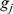. Note also that for the same reason, once trained, the system works fast, not slowly.

In the computationally relevant case where 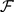 is finite-dimensional the solution to the optimization problem can be found by means of Slow Feature Analysis (SFA) [26], [27]. This algorithm, which is based on an eigenvector approach, is guaranteed to find the global optimum. Biologically more plausible learning rules for the optimization problem exist [28], [29].

### Hierarchical network model

The visual system is, to a first approximation, structured in a hierarchical fashion, first extracting local features which are then integrated to more and more global and abstract features. We apply SFA in a similar hierarchical manner to the raw visual input data. First, the slow features of small local image patches are extracted. The integration of spatially local information exploits the local correlation structure of visual data. A second layer extracts slow features of these features (again integrating spatially local patches), and so on. Such hierarchical architecture is promising because SFA has been applied successfully to visual data in a hierarchical fashion previously [15], [30]. A hierarchical organization also turns out to be crucial for the applicability of the approach for computational reasons. The application of non-linear SFA on the whole high-dimensional input would be computationally infeasible. Efficient use of resources is also an issue in biological neural circuits. It has been suggested that connectivity is the main constraint there [31], [32]. Since a hierarchical organization requires nearly exclusively local communication, it avoids extensive connectivity.

The hierarchical network consists of a converging hierarchy of layers of SFA nodes, and the network structure is identical to that used in [30] (there this part of our model is also discussed in greater length). All required building blocks for the hierarchical network are available in the “Modular toolkit for Data Processing” (MDP) library [33].

#### Network structure

**Figure 3:**
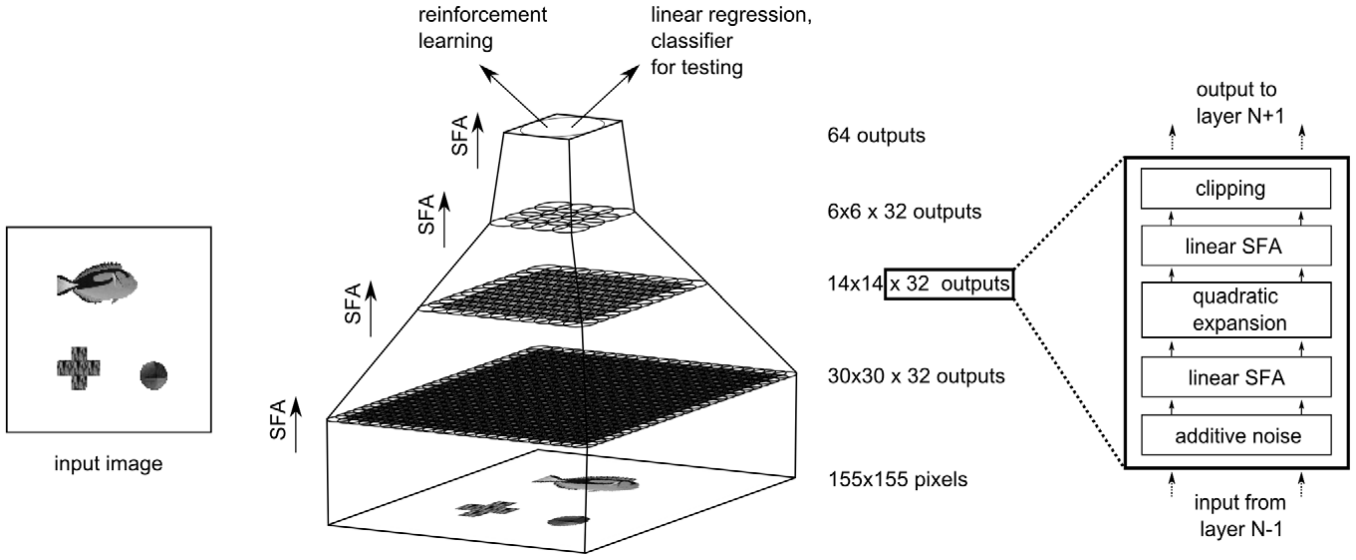
Model architecture and stimuli. An input image is fed into the hierarchical network. The circles in each layer symbolize the overlapping receptive fields, which converge towards the top layer. The same set of steps is applied on each layer, which is visualized on the right hand side.

The detailed network structure is shown in Figure 3. It consists of four layers of SFA nodes, connected topographically in a feed-forward manner. We first describe the internal organization of each individual SFA node before we give a detailed description of the connection architecture below. In each SFA node, first additive Gaussian white noise (with a variance of 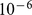) is introduced for numerical reasons, to avoid possible singularities in the subsequent SFA step. Then a linear SFA is performed for a first reduction of the input dimensionality. In a subsequent quadratic expansion, the incoming data 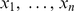 is mapped with a basis of the space of polynomials with degree up to two. So in addition to the original data, all quadratic combinations like 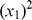 or 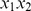 are concatenated to the data block. Another linear SFA stage is applied on the expanded data. The solutions of linear SFA on this expanded data is equivalent to those of SFA in the space of polynomials up to degree two. After the second SFA stage we apply a clipping at 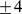. This clipping removes extreme values that can occur on test data due to the divergence of the quadratic functions for larger values. However, both the additive noise and the clipping are mostly just technical safeguards and have typically no effect on the network performance.

The number of SFA components used from the first linear SFA stage in each node depends on the layer in which the SFA node is situated. The first linear SFA stage in each node reduces the dimensionality to 32 in the first two layers, 42 in the third layer, and 52 in the fourth layer (the increase in dimensionality across layers leads to a small performance increase). Accordingly, the quadratic expansion then increases dimensionality to 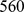, 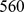, 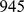 and 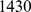, in the first, second, third, and fourth layer respectively. The second linear SFA stage reduces the dimensionality of the expanded signal to 32, except for the top layer, where the output is reduced to 64 dimensions. One can then choose how many of these outputs are actually used in the reinforcement learning (for the variable-targets task the 32 slowest outputs were used).

**Figure 4:**
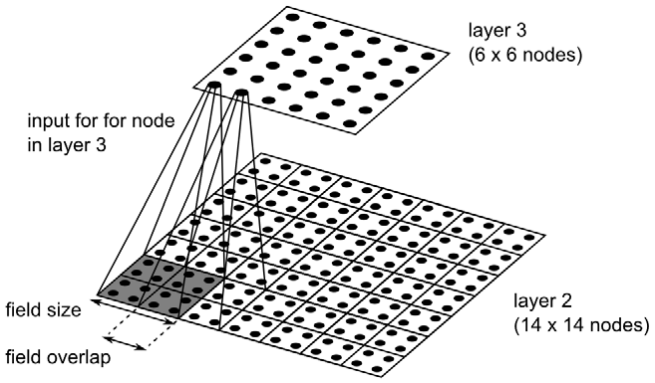
Receptive field of nodes in layer 3. Each dot represents the 32 dimensional SFA output from one node. The field overlap is 2 nodes and the borders of the receptive fields are represented by the black lines between the dots.

We now describe how the nodes are connected (see Figure 4). We use a layered feed-forward architecture, i.e., the nodes in the first layer receive inputs only from the input image and nodes in higher layers receive inputs exclusively from the previous layer. Additionally, connections are topographically structured such that a node receives inputs from neighboring nodes in the previous layer. In the following, the part of the input image that influences a node's output is denoted as its receptive field. On the lowest layer, the receptive field of each node consists of an image patch of 10 by 10 grayscale pixels. The receptive fields jointly cover the input image of 155 by 155 pixels. The nodes form a regular (i.e., non-foveated) 30 by 30 grid with partially overlapping receptive fields, resulting in an overlap of five pixels in each direction. The second layer contains 14 by 14 nodes, each receiving input from 4 by 4 layer 1 nodes with neighboring receptive fields, resembling a retinotopic layout (the overlap is two nodes in each direction). The third layer contains 6 by 6 nodes, each receiving input from 4 by 4 layer 2 nodes with neighboring receptive fields, again in a retinotopic layout (with 2 nodes overlap in each direction, as shown in Figure 4). All 6 by 6 layer 3 outputs converge onto a single node in layer 4, whose output we call SFA-output. This organization is summarized in Table 1.

**Table 1:** Overview of the network architecture.

Layer	Number of nodes	Input area of node	Overlap per direction	SFA outputs per node
0 (Image)	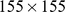	-	-	(1 pixel)
1	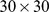	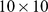	5	32
2	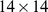	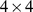	2	32
3	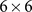	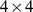	2	32
4	1	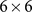	-	64

Layer 0 denotes the input image, a node corresponds to a pixel in that image. The input area denotes the number of nodes in the previous layer from which a node receives input, this is also called the receptive field. An example for layer 3 is visualized in Figure 4.

Thus, the hierarchical organization of the model captures two important aspects of cortical visual processing: increasing receptive field sizes and accumulating computational power at higher layers. The latter is due to the quadratic expansion in each layer, so that each layer computes a subset of higher polynomials than its predecessor. The SFA-outputs at the top layer compute subsets of polynomials of degree 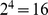.

#### Network training

For each of the two tasks discussed in this paper (Morris water-maze and variable-targets) we trained a dedicated hierarchical network. The number of training samples and the training itself was done in the same way for both tasks, only the content of the training samples was different (this is described in the next section).

The network layers were trained sequentially from bottom to top. We used 50,000 time points for the training of the two lower layers and 200,000 for the two top layers. These training sequences were generated with a random walk procedure, which is described in the next section. The random walk parameters of the training data were identical for all layers. The larger training set for the top layers is motivated by the smaller multiplicative effect of the weight-sharing and by the slower time scales towards the top (though one has to combine this factor with the complexity of the data structure).

For computational efficiency, we train only one node with stimuli from all node locations in its layer and replicate this node throughout the layer. For example this means that the node in the lowest layer sees 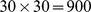 times as much data as if it was only trained at a single location. This mechanism effectively increases the number of training samples and implements a weight-sharing constraint. However, the system performance does not depend on this mechanism. The statistics of the training data are approximately identical for all receptive fields, so individually learned nodes would lead to the same results (but at higher computational cost). While the weight-sharing does ease the emergence of translation invariance it is not at all sufficient.

The simulated views are generated from their configuration (position, angles, and object identity) with floating point precision and are not artificially discretized.

#### Training stimuli of the hierarchical network

The training sequences for the two tasks were created with the same random walk procedure that was used in [30]. The configuration of the objects shown (i.e. the agent in the water-maze task, and for the variable-targets task also target and distractor) was updated in each timestep. Such an update consists of adding a random term to the current spatial velocities of the objects and to the in-plane angular velocity for the agent object (the fish). The velocities are then used to calculate the new positions of the objects, which are in the interval 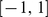, and the new angle of the agent. The velocity distribution was the same for all objects (max. velocity of 0.06 and a max. update of 0.01). For the in-plane angle of the agent the max. velocity was 0.04 with a max. update of 0.01 (in radiant measure).

For the variable-targets task training the objects were given a radius so that they bounce off each other. The radii were chosen such that there could be only a small visible overlap between any two objects (radius of 0.4 for the agent, 0.2 for target and distractor). In each time step the agent identity was switched with a probability of 0.002.

### Neural circuits for reward-based learning

We employed neural implementations of two reinforcement learning algorithms, one is based on Q-learning and one is a policy-gradient method.

Neural versions of Q-learning have been used in various previous works on biological reward-based learning, see e.g. [34], [35]. The popularity of Q-learning stems from the finding that the activity of dopaminergic neurons in the ventral tegmental area is related to the reward-prediction error [4], [36], [37], a signal that is needed in Q-learning [35]. In Q-learning, decisions are based on a so-called Q-function that maps state-action pairs 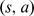 onto values that represent the current estimate of the expected total discounted reward given that action 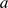 is executed at state 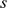. For a given state, the action with highest associated Q-value is preferred by the agent. However, to ensure exploration, a random action may be chosen with some probability. We implemented the neural version of Q-learning from [35] where the Q-function is represented by a small ensemble of neurons and parametrized by the connection weights from the inputs to these neurons. The system learns by adaptation of the Q-function via the network weights. In the implementation used in this article, this is achieved by a local synaptic learning rule at the synapses of the neurons in the neuron ensemble. The global signal that modulates local learning is the temporal difference error (TD-error). We do not address in this article the question how this signal is computed by a neuronal network. Several possible mechanisms have been suggested in the literature [37]–[39].

The Q-function was represented by a set of 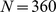 linear neurons that receive information about the current state from the output 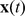 of the SFA circuit. The output 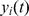 of neuron 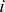 is hence given by 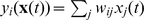.

Each neuron 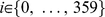 has a dedicated preferred direction 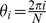. The Q-value 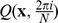 of a movement in direction 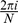 for the given state 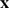 is hence given by 
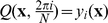
. The activities of these neurons imply a proposed action for the agent which is a movement in the direction given by the population vector 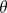. Here, 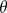 is the angle of the vector
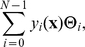
(5)where the vector 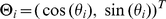 is the unit vector in direction 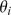.

The Q-function is parametrized by the weight values 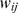 and it is learned by adapting these weights according to the Q-learning algorithm (see [35]):

1. For time step 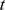, compute the Q-values 
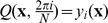
.
2. Let 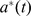 be a movement in direction of the population vector 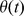

3. Choose the next action 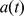 to be 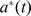 with probability 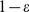 or a movement in a random direction with probability 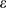.
4. A Gaussian profile around the chosen action 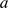 is enforced in the neural ensemble resulting in 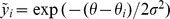.
5. The eligibility trace is updated according to 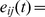 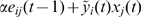.
6. Action 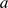 is executed and time is updated 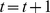.
7. The reward prediction error is calculated as 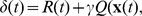 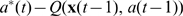.
8. Update the weights of the neuron population according to 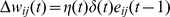 with 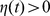 being a small decaying learning rate.

See Supporting Text S1 for parameter settings.

The second learning algorithm employed was a policy gradient method. In this case, the action is directly given by the output of a neural network. Hence, the network (which receives as input the state-representation from the SFA network) represents a policy (i.e., a mapping from a state to an action). Most theoretical studies of such biologically plausible policy-gradient learning algorithms are based on point-neuron models where synaptic inputs are weighted by the synaptic efficacies to obtain the membrane voltage. The output 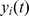 of the neuron 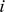 is then essentially obtained by the application of a nonlinear function to the membrane voltage. A particularly simple example of such a neuron model is a simple pseudo-linear rate-based model where a nonlinear activation function 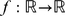 (commonly sigmoidal) is applied to the weighted sum of inputs 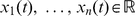:
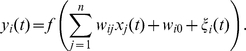
(6)Here, 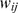 denotes the synaptic efficacy (weight) of synapse 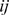 that projects from neuron 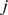 to neuron 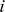, 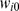 is a bias, and 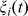 denotes some noise signal. We assume that a reward signal 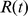 indicates the amount of reward that the system receives at time 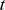. Good actions will be rewarded, which will lead to weight changes that in turn make such actions more probable. Reinforcement learning demands exploration of the agent, i.e., the agent has to explore new actions. Thus, any neural system that is subject to reward-based learning needs some kind of stochasticity for exploration. In neuron model (6) exploration is implemented via the noise term 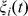. Reward-based learning rules for this model can easily be obtained by changing the weights in the direction of the gradient of 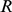


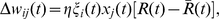
(7)where 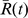 denotes the low-pass filtered version of 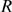 with an exponential kernel, and 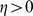 is a small learning rate. In our simulations we used 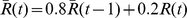 for the filtered reward. The update equations for the bias is analogous with 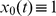.

A single neuron of type (6) turns out to be too weak for some of the control tasks considered in this article. The standard way to increase the expressive power is to use networks of such neurons. The learning rule for the network is then unchanged, each neuron tries to optimize the reward independently from the others [40], but see [41]. It can be shown that such a greedy strategy still performs gradient ascent on the reward signal. However, the time needed to converge to a good solution is often too long for practical applications as shown in Results. We therefore propose a learning rule that is based on a more complex neuron model with nonlinear dendritic interactions within neurons [42] and the possibility to adapt dendritic conductance properties [43].

In this model, the total somatic input 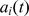 to neuron 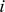 is modeled as a noisy weighted linear sum of signals from dendritic branches
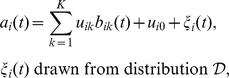
(8)where 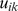 describes the coupling strength between branch 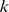 and the soma and 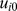 is a bias. Again, 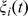 models exploratory noise. At each time step, an independent sample from the zero mean distribution 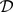 is drawn as the exploratory signal 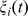. In our simulations, 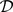 is the uniform distribution over the interval 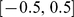. The output 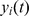 of neuron 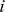 at time 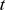 is modeled as a nonlinear function of the total somatic input:
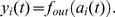
(9)Each dendritic branches 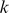 itself sums weighted synaptic inputs followed by a sigmoidal nonlinearity 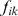


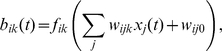
(10)where 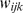 denotes the synaptic weight from input 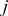 to the dendritic branch 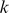 of neuron 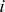. Update equations that perform gradient ascent on a reward-signal 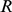 are derived in Supporting Text S2. The derived update rules for the parameters are
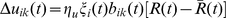
(11)

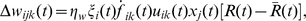
(12)where and 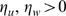 are small learning rates. The update rules can be extended to use eligibility traces that collect the information about recent pre-and postsynaptic states at the synapse in a single scalar value. In this way, previous states of the synapse can be incorporated in the weight change at time 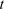, which is driven by the momentary reward signal 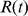. In this article however, we rely on the update rules (11) and (12) without eligibility traces. See [41] for an alternative rule of similar flavor.

In our simulations, we needed two control variables, one to control the speed 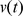 of the agent and one for its angular velocity 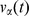. Each control variable was computed by a single neuron of this type where each neuron had 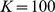 branches. The nonlinearity in the branches was the tangens hyperbolicus function 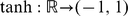. Also a logistic sigmoidal was tested which is a scaled version of the tangens hyperbolicus to the image set 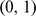. Results were similar with a slight increase in learning time. The nonlinearity at the soma was the tangens hyperbolicus for the angular velocity 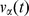 and a logistic sigmoid 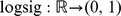 for the speed 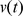. The noise signal 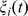 was drawn independently for each neuron and at each time step from a uniform distribution in 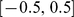. Detailed parameter settings used for the simulations can be found in Supporting Text S1.

### Tasks

We tested the system on two different control tasks: a task similar to the Morris water-maze task and a variable-targets task.

#### Morris water maze task

In the experimental setup of a Morris water maze task [25], a rat swims in a milky liquid with a hidden platform underneath the liquid surface. Because the rodent tries to avoid swimming in the liquid, it searches for the platform. This task has been modeled several times [34], [35], [44], [45].

In order to be able to compare the results to previous studies, we modeled the Morris water maze task in our standard setup in the following way: We used only a single fish type and a fixed target position at 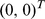. Only the fish but not the target was visible in the visual input to the learning system. There was only one control signal which controlled the direction of the next movement. At each time step, the fish was moved by 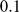 length units in the direction given by the controller. The position of the fish was hard-bounded by 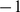 from below and 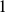 from above after each update such that it stayed within 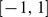. In this setup, the fish was always oriented in same direction (facing to the right), i. e., the fish was not rotated in the visual input. The target was reached by the agent if it was within a radius of 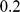 of the target position.

The reward signal was defined such that reaching the target at time 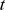 resulted in a positive reward 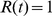, hitting the wall at time 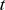 resulted in a negative reward 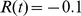, and the reward signal was 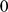 at other times. Hence this is a setup with sparse rewards. An episode ended when the target was reached or after 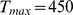 time-steps have evolved (this is consistent with [44] where a simulation time step was interpreted as a 200 msec time interval).

#### Variable-targets task

In order to explore the general applicability of the system we investigated a more demanding task with several objects in the visual input and two different types of fish of varying orientation.

In this task, the state of the agent at time 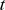 was defined by its identity 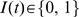 (this corresponds to two types of fish, each with a unique visual appearance in the visual input stream), its position 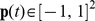, and its orientation 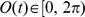. Additionally to the agent, there were two objects in the universe, one of them acting as the target and the other as a distractor. One object had appeared as a “cross” in the visual scene and the other object as a “disk” (see Figure 2). The state of object 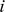 was defined by its position 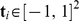. The current state of the universe at time 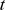 was given by the collection of these variables.

The output of the learning system were two control variables to control the agent in the environment, a speed signal 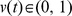 and a signal 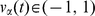 for angular velocity. These signals were used to update the orientation 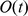 and position 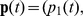 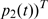 of the fish
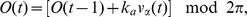
(13)

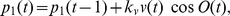
(14)

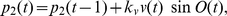
(15)where 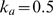 and 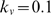 are scaling constants. When the agent hit the boundaries of the environment (i.e., when 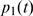 or 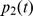 were below 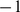 or above 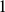), the movement was mirrored. For each training episode, object positions, fish orientation, and fish identity were initially chosen randomly from the uniform distribution in their range. However, when an object was less than 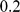 away from the other object or the fish (which likely produced a visual overlap), a new initial state was drawn. The object positions were then fixed. Each fish identity had a different object serving as the target, such that the fish of type A was associated with the “cross” whereas fish-type B was associated with the “disk”. The task was to navigate the fish to the target object for the given fish identity. The current episode ended when the fish reached the target location within some predefined radius (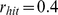) or after a maximum of 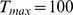 time steps were exceeded). Although the other object did not influence the outcome of the task, it was still visible as a distracting stimulus.

The reward signal indicated whether the last action was successful in bringing the agent closer to the target:
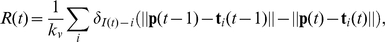
(16)where 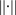 denotes the Euclidean norm and 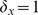 if 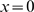 and 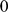 otherwise. This is a relatively informative reward signal (see Discussion).

## Results

### Morris water maze task

**Figure 5:**
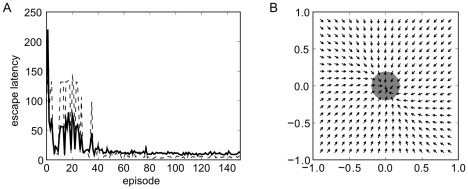
Performance of the learning system in the Morris water maze task with Q-learning. A) Mean escape latency (in simulation time steps) as a function of learning episodes for 10 independent sets of episodes (full thick line). The thin dashed line indicates the standard deviation. B) The navigation map of the system after training. The vectors indicate the movement directions the system would most likely choose at the given positions in the water maze. An episode ended successfully when the center of the fish reached the area indicated by the gray disk.

We implemented this task with our learning system where the decision circuit consisted of the Q-learning circuit described above. In this task, the 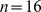 slowest components as extracted by the hierarchical SFA network were used by the subsequent decision network. The results of training are shown in Figure 5. The performance of the system was measured by the time needed to reach the target (escape latency). The system learns quite fast with convergence after about 40 training episodes. The results are comparable to previously obtained simulation results [34], [35], [44] that were based on a state representation by neurons with place-cell-like behavior. Figure 5B shows the direction the system chooses with high probability at various positions in the water maze (navigation map) after training. Using only the 16 slowest SFA components for reinforcement learning, the system has rapidly learned a near-optimal strategy in this task. This result shows that the use of SFA as preprocessing makes it possible to apply reinforcement learning to raw image data in the Morris water maze task.

### Variable-targets task

The Morris water maze task is relatively simple and does not provide rich visual input. We therefore tested the learning system on the variable-targets task described above, a control task where two types of fish navigate in a 2D environment. In the environment, two object positions were marked by a cross and a disk, and these positions were different in each learning episode. A target object was defined for each fish type and the task was to navigate the current fish to its target by controlling the forward speed and the change in movement direction (angular velocity). The control of angular velocity, the arbitrary target position, and the dependence of the target object on the fish identity complicates the control task such that the Q-learning algorithm used in the water-maze task as well as a simple linear decision neuron like the one of equation (6) would not succeed in this task. We therefore trained the leaning system with the more powerful policy gradient algorithm described above on the slowest 32 components extracted by the hierarchical SFA network.

In order to compute the SFA output fast, we had to perform the training of the control network in batches of 100 parallel traces in this task (i.e., 100 training episodes with different initial conditions are simulated in parallel with a given weight vector. After the simulation of a single time step in all 100 episodes, weight changes over these 100 traces are averaged and implemented. Then, the next time step in each of the 100 traces is simulated and weights are updated). When the agent in one of the traces arrived at the target, a new learning episode was initiated in this trace while other traces simply continued. As will be shown below, the training in batches has no significant influence on the learning dynamics.

**Figure 6:**
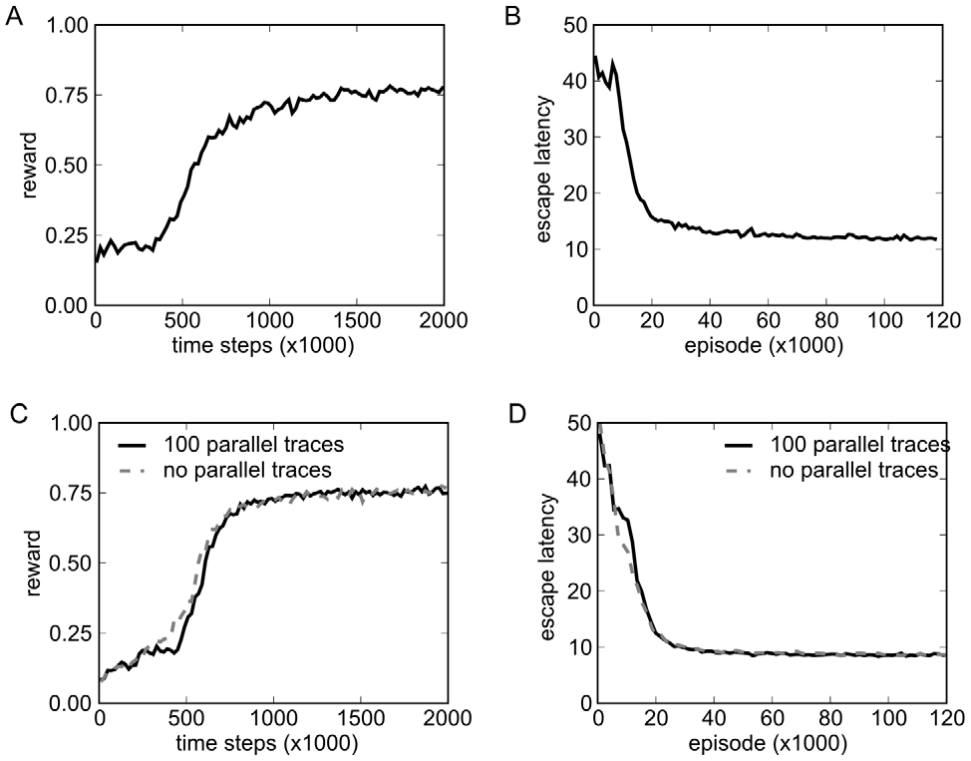
Rewards and escape latencies during training of the control task with target and distractor. A) Evolution of reward during training. A simulation step for all 100 parallel traces corresponds to 100 time-steps at the x-axis. The plotted values are averages over consecutive 20,000 time steps. B) Evolution of escape latencies (measured in time steps) during training. The number of episodes on the x-axis is the number of completed traces. The plotted values are averages over 1,200 consecutive episodes. C,D) Same as panels A and B, but learning was performed on a highly condensed and precise state-encoding instead of the SFA network output. Shown is the performance for learning on 100 parallel traces (black, full line) and without parallel traces (gray, dashed line). Convergence is comparable to learning on SFA outputs. The results without parallel traces are very similar to the results with parallel traces.

**Figure 7:**
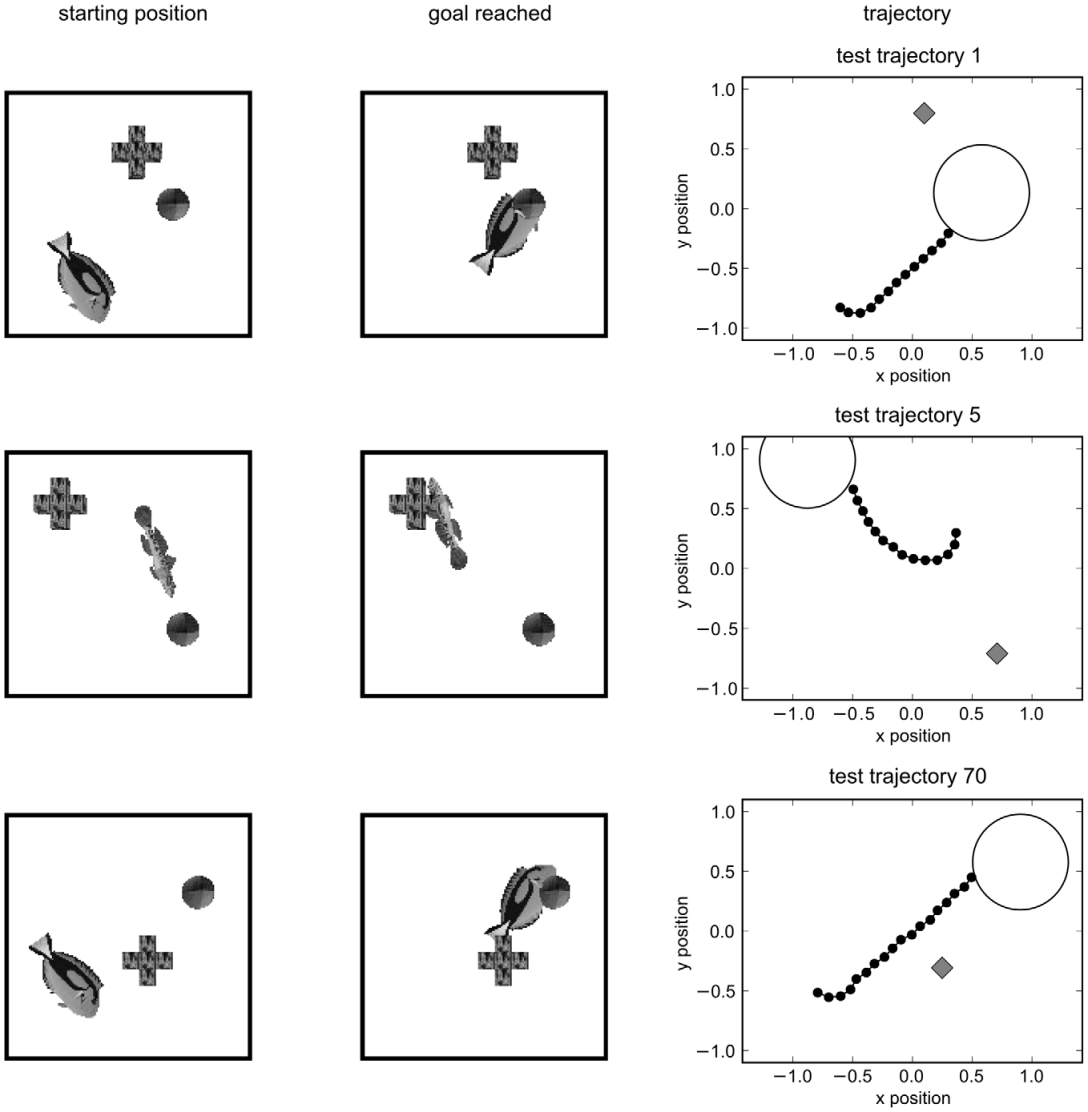
Three representative trajectories after training of the control task with target and distractor. Each row summarizes one representative learning trial. Shown is the visual input at start position (left column), the visual input when the goal was reached (middle column), and the whole trajectory (right column). In the trajectory, fish positions (small black discs), target region (large circle), and distractor location (gray rectangle) are shown.

Results are shown in Figure 6A,B. The reward converges to a mean reward above 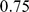 which means that the agent nearly always takes the best step towards the target despite the high amount of noise in the control neurons. Figure 7 shows that the trajectories after training were very good. Interestingly, the network does not learn the optimal strategy with respect to the forward speed output. Although it would be beneficial to reduce the forward speed when the agent is directed away from the target, first rotate the agent, and only then move forward, the output of the speed neuron is nearly always close to the maximum value. A possible reason for this is that the agent is directed towards the target most of the time. Thus, the gain in reward is very small and a relatively small fraction of training examples demands low speed.

We compared the results to a learning system with the same control circuit, but with SFA replaced by a vector which directly encoded the state-space in a straight-forward way. For this task with two fish identities and two objects, we encoded the state-space by a vector
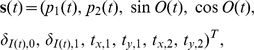
(17)where 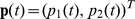 is the position of the agent, 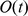 is its orientation, 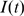 is its identity, and 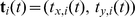 is the position of the 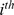 object. Figure 6C,D shows the results when the control network was trained with identical parameters but with this state-vector as input. The Performance with the SFA network is comparable to the performance of the system with a highly informative and precise state encoding.

For efficiency reasons, we had to perform the training of the control network in batches of 100 traces (see above). Because no SFA is needed in the setup with the direct state-vector as input, we can compare learning performance of the control network to performance without batches. The result is shown in in Figure 6C,D (gray dashed lines). The use of small batches does not influence the learning dynamics significantly.

In the environment considered, movement is mirrored if the agent hits a boundary. Since this helps to avoid getting stuck in corners we performed control experiments where the movement in the direction of the boundary is simply cut off but no reflection happens (i.e., the dynamics of the position 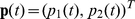 of the fish is given by 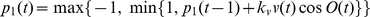 and 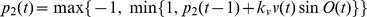, compare to equations (14),(15)). Results are shown in Figure S1. As expected, the system starts with lower performance and convergence takes about twice as long compared to the environment with mirrored movements at boundaries. Interestingly, in this slightly more demanding environment, the SFA network is converging faster than the system with a highly informative and precise state encoding.

In another series of experiments we tested how the performance depends on the number of outputs from the SFA network that are used as input for the reinforcement learning. Since the outputs of the SFA network are naturally ordered by their slowness one can pick only the first 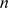 outputs and train the reinforcement learning network on those. For the variable-targets task we tested the performance for 16, 22, 28, 32, and 64 outputs. For 16 outputs the average reward value always stayed below 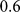 and rose much slower than in the case of 32 outputs. For 28 outputs the performance was already very close to that of the 32 outputs. Going from 32 outputs to 64 did not change the average reward, but in the case of 64 outputs the trajectories of the agent occasionally showed some errors (e.g., the agent initially chose a wrong direction and took therefore longer to reach the target).

We compared performance of the system to a system where the control network is a two-layer feed-forward network of simpler neurons without dendritic branches, see Equation (6). We used two networks with identical architecture, one for each control variable. Each network consisted of 50 neurons in the first layer connected to one output neuron (increasing the number of neurons in the first layer to 100 did not change the results). Every neuron in the first layer received input from all SFA outputs. The learning rates of all neurons were identical. See Supporting Text S1 for details on parameters and their determination. Results are shown in Figure S2. The network of simple neurons can solve the problem in principle, but it converges much slower.

**Figure 8:**
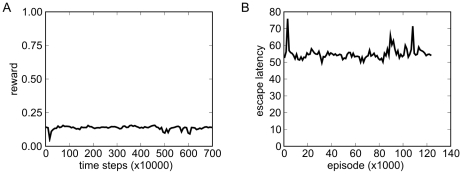
Performance of a PCA based hierarchical network. Rewards (A) and escape latencies (B) in the variable-targets control experiment with a PCA based hierarchical network. The control network is not able to learn the task based on this state representation. Note the larger scaling factor for the time-axis in panel A.

We also compared performance of the system with SFA to systems where the dimensionality of the visual input was reduced by PCA. In one experiment the SFA nodes in the hierarchical network were simply replaced by PCA nodes. We then used 64 outputs from the network for the standard reinforcement learning training. As shown in Figure 8 the control network was hardly able to learn the control task. This is also evident in the test trajectories, which generally look erratic.

In another experiment we used PCA on the whole images. Because of the high dimensionality we first had to downsample the image data by averaging over two by two pixels (reducing the dimensionality by a factor of four) before using linear PCA. The performance was very similar to the hierarchical PCA experiment (the average reward hovered below 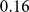). A direct analysis of the PCA output with linear regression [15] indicates that except for the agent identity, no important features such as position of the agent or the targets can be extracted in a linear way from the reduced state representation. For hierarchical SFA, such an extraction is often possible [15]. This hints at the possibility that the state representation given by PCA cannot be exploited by the control network because the implicit encoding of relevant variables is either too complex or too much important information has been discarded.

## Discussion

Several theoretical studies have investigated biologically plausible reward-based learning rules [46]–[55]. On the synaptic level, such rules are commonly of the reward-modulated Hebbian type, also called three-factor rules. In traditional Hebbian learning rules, changes of synaptic plasticity at time 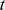 are based on the history of the presynaptic and the postsynaptic activity, such that the weight change 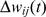 of a synapse from a presynaptic neuron 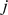 to a postsynaptic neuron 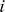 is the product between some function of the presynaptic activity history and some function of the postsynaptic activity history. A third signal 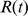 that models the local concentration of some neuromodulator which in turn signals some reward, is in many models modulating these Hebbian updates. Such update rules are either purely phenomenological [53], [55] or derived from a reward-maximization principle [47]–[51]. From the viewpoint of classical reinforcement learning, the latter approach is related to policy-gradient methods. Since the learning algorithms in these previous works are based on simple neuron models, they are too weak for the variable-targets task considered in this article. The policy-gradient method used in this article extends the classical single-neuron based policy-gradient approach in the sense that it is based on a more expressive neuron model with nonlinear branches. In this model, both, synaptic weights and branch strengths are adapted through learning. Our approach is motivated by recent experimental findings where it has been shown that not only synaptic efficacies but also the strengths of individual dendritic branches are plastic [43]. Furthermore, it was shown that this type of plasticity is dependent on neuromodulatory signals. Our results (compare Figure 6 to Figure S2) indicate that the neuron model with nonlinear branches can be trained much faster than networks of point-neuron models. This hints at a possible role of nonlinear branches in the context of reward-based learning.

The Morris water-maze task has been modeled before. In [45], a network of spiking neurons was trained on a relatively small discrete state-space that explicitly coded the current position of the agent on a two-dimensional grid. The authors used a neural implementation of temporal difference learning. In contrast to the algorithms used in this article, their approach demands a discrete state space. This algorithm is therefore not directly applicable to the continuous state-space representation that is achieved through SFA. In [34] and [44] the input to the reinforcement learning network was explicitly coded similar to the response of hippocampal place-cells. In [35], the state-representation was also governed by place-cell-like response that were learned from the input data. This approach was however tailored to the problem at hand, whereas we claim that SFA can be used in a much broader application domain since it is not restricted to visual input. Furthermore, in this article SFA was not only used to extract position of an agent in space but also for position of other objects, for object identity, and for orientation. We thus claim that the learning architecture presented is very general only relying on temporal continuity of important state variables.

Although the variable-targets task considered above is quite demanding, the learning system gets immediate feedback of its performance via the reward signal defined by equation (16). By postulating such a reward signal one has to assume that some system can evaluate that “getting closer to the target” is good. Such prior knowledge could have been acquired by earlier learning or it could be encoded genetically. An example of a learning system that probably involves such a circuitry (the critique) is the song-learning system in the songbird. In this system, it is believed that a critique can evaluate similarity between the own song and a memory copy of a tutor song [56]. However, there is no evidence that such higher-level critique is involved for example in navigational learning of rodents. Instead, it is more natural to assume that an internal reward signal is produced for example when some food-reward is delivered to the animal. One experimental setup with sparse rewards is the Morris water maze task [25] considered above. In principle, this sparse reward situation could also be learned if the learning rules (11), (12) are amended with eligibility traces [48]. However, the learning would probably take much longer.

Given the high-dimensional visual encoding of the state-space accessible to the learning system, it is practically impossible that any direct reinforcement learning approach is able to solve the variable-targets task directly on the visually-induced state-space. Additionally, in order to scale down the visual input to viable sizes, a hierarchical approach is most promising. Here, hierarchical SFA is one of the few approaches that have been proven to work well. Linear unsupervised techniques such as principal component analysis (PCA) or independent component analysis (ICA) are less suited to be applied hierarchically. To understand the results, it is important to note that SFA is quite different from PCA or other more elaborate dimensionality reduction techniques [57], [58]. Dimensionality reduction in general tries to produce a faithful low-dimensional representation of the data. The aim of SFA is not to produce a faithful representation in the sense that the original data can be reconstructed with small error. Instead, it tries to extract slow features by taking the temporal dimension of the data into account (this dimension is not exploited by PCA) and disregards many details of the input. Although it is in general not guaranteed that slowly varying features are also important for the control task, slowly varying features such as object identities and positions are important in many tasks. In fact, the removal of details may underlie the success of the generic architecture, since it allows the subsequent decision circuit to concentrate on a few important features of the input. This may also explain the failure of PCA. The encoding of the visual input produced by PCA can be used to reconstruct a “blurred” version of the input image. However, it is very hard to extract from this information the relevant state variables such as object identity or position. But this information can easily be extracted from the SFA output, see [15].

We compared the preprocessing with SFA to PCA preprocessing but not to more elaborate techniques [57], [58] since the focus of this paper is on simple techniques for which some biological evidence exists. Another candidate for sensory preprocessing instead of SFA is ICA. However, ICA does not provide a natural ordering of extracted components. It is thus not clear which components to disregard in order to reduce the dimensionality of the sensory input stream. One interesting possibility would be to order the ICA components by kurtosis in order to extract those components which are most non-Gaussian. Another interesting possibility not pursued in this paper would be to sparsify the SFA output by ICA. This has led to place-cell like behavior in [14] and might be beneficial for subsequent reward-based learning. Information bottleneck optimization (IB) is another candidate learning mechanism for cortical feature extraction. However, IB is not unsupervised, it needs a relevance signal. It would be interesting to investigate whether a useful relevance signal could be constructed for example from the reward signal. Finally, the problem of state space reduction has also been considered in the reinforcement learning literature. There, the main approach is either to reduce the size of a discrete state space or to discretize a continuous state-space [59],[60]. In contrast, SFA preserves the continuous nature of the state-space by representing it with a few highly informative continuous variables. This circumvents many problems of state-space discretization such as the question of state-space granularity. Thus, there are multiple benefits of SFA in the problem studied: It can be trained in a fully unsupervised manner (as compared to IB). By taking the temporal dimension into account, it is able to compress the state-space significantly without the need to discretize the continuous state-space (as compared to [59], [60]). It provides a highly abstract representation that can be utilized by simple subsequent reward-based learning (compare to the discussion of PCA). The possibility to apply SFA in a hierarchical fashion renders it computationally efficient even on high-dimensional input streams, both in conventional computers and in biological neural circuits where it allows for mainly local communication and thus avoids extensive connectivity [31], [32]. The natural ordering of features based on their slowness implies a simple criterion on the basis of which information can be discarded in each node of the hierarchical network (compare to ICA), resulting in a significant reduction of information that has to be processed by higher-level circuits. Finally, SFA is relatively simple, its complexity is comparable to PCA and it is considerably simpler than other approaches for state-space reduction [57]–[60]. Accordingly, biologically plausible implementations of SFA exist [28], [29]. Together with the fact that experimental evidence for slowness learning exists in the visual system [23], this renders SFA an important candidate mechanism for unsupervised feature extraction in sensory cortex.

In this article, we provided a proof of concept that a learning system with an unsupervised preprocessing and subsequent simple biologically realistic reward-based learning can learn quite complex control tasks on high-dimension visual input streams without the need for hand-design of a reduced state-space. We applied the proposed learning system to two control tasks. In the Morris water maze task, we showed that the system can find an optimal strategy in a number of learning episodes that is comparable to experimental results with rats [25]. The application of the learning system to the variable targets task shows that also much more complex tasks with rich visual inputs can be solved by the system. We propose in this article that slowness-learning in combination with reward-based learning may provide a generic (although not exclusive) principle for behavioral learning in the brain. This hypothesis predicts that slowness learning should be a major unsupervised learning mechanism in sensory cortices of any modality. Currently, such evidence exists for the visual pathway only [23]. We showed that learning performance of the system in this task is comparable to a system where the state-representation extracted by SFA is replaced by a highly compressed and precise hand-crafted state-space. Finally, our simulation results suggest that performance of the system is quite insensitive to the number of SFA components that is chosen for further processing by the reinforcement learning network as long as enough informative features are chosen.

Altogether this study provides, on the one hand, further support that slowness learning could be one important (but not necessarily exclusive) unsupervised learning principle utilized in the brain to form efficient state representations of the environment. On the other hand, this work shows that autonomous learning of state-representations with SFA should be further pursued in the search for autonomous learning systems that do not - or much less - have to rely on expensive tuning by human experts.

## Supporting Information

Figure S1 Rewards and escape latencies during training of the control task with target and distractor without mirrored movements at boundaries. A) Evolution of reward during training. A simulation step for all 100 parallel traces corresponds to 100 time-steps at the x-axis. The plotted values are averages over consecutive 50,000 time steps. B) Evolution of escape latencies (measured in time steps) during training. The number of episodes on the x-axis is the number of completed traces. The plotted values are averages over 3,000 consecutive episodes. C,D) Same as panels A and B, but learning was performed on a highly condensed and precise state-encoding instead of the SFA network output. Shown is the performance for learning on 100 parallel traces (black, full line) and without parallel traces (gray, dashed line). Convergence is slower compared to learning on SFA outputs.
(0.02 MB PDF)

Figure S2 Rewards and escape latencies during training of a feed-forward network of simple neurons on the control task with target and distractor. A) Evolution of reward during training. A simulation step for all 100 parallel traces corresponds to 100 time-steps at the x-axis. The plotted values are averages over consecutive 150,000 time steps. B) Evolution of escape latencies (measured in time steps) during training. The number of episodes on the x-axis is the number of completed traces. The plotted values are averages over 8,000 consecutive episodes.
(0.02 MB PDF)

Text S1 Detailed parameters for reward-based learning.
(0.02 MB PDF)

Text S2 Derivation of the policy-gradient update rule.
(0.02 MB PDF)
