# Supplementary material for: Reinforcement Learning on Slow Features of High-Dimensional Input Streams
Source: PLoS Comput Biol. 2010 Aug 19;6(8):e1000894. doi: 10.1371/journal.pcbi.1000894 (PMC2924248; doi:10.1371/journal.pcbi.1000894)
Supplement: Figure S1 — Rewards and escape latencies during training of the control task with target and distractor without mirrored movements at boundaries. A) Evolution of reward during training. A simulation step for all 100 parallel traces corresponds to 100 time-steps at the x-axis. The plotted values are averages over consecutive 50,000 time steps. B) Evolution of escape latencies (measured in time steps) during training. The number of episodes on the x-axis is the number of completed traces. The plotted values are averages over 3,000 consecutive episodes. C,D) Same as panels A and B, but learning was performed on a highly condensed and precise state-encoding instead of the SFA network output. Shown is the performance for learning on 100 parallel traces (black, full line) and without parallel traces (gray, dashed line). Convergence is slower compared to learning on SFA outputs. (0.02 MB PDF) [file pcbi.1000894.s001.pdf]

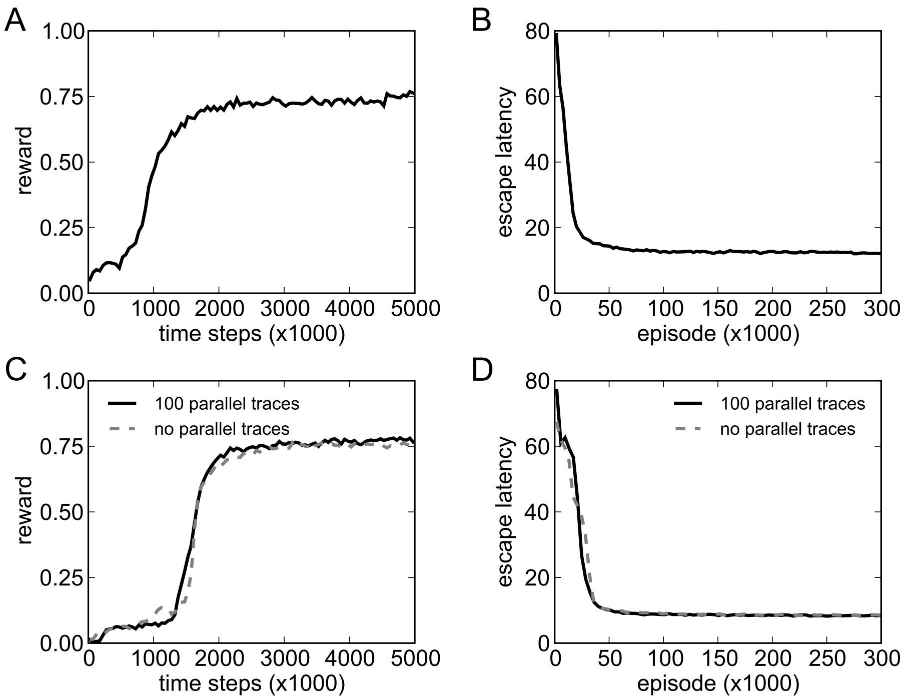

Figure S1: **Rewards and escape latencies during training of the control task with target and distractor without mirrored movements at boundaries.** A) Evolution of reward during training. A simulation step for all 100 parallel traces corresponds to 100 time-steps at the x-axis. The plotted values are averages over consecutive 50,000 time steps. B) Evolution of escape latencies (measured in time steps) during training. The number of episodes on the x-axis is the number of completed traces. The plotted values are averages over 3,000 consecutive episodes. C,D) Same as panels A and B, but learning was performed on a highly condensed and precise state-encoding instead of the SFA network output. Shown is the performance for learning on 100 parallel traces (black, full line) and without parallel traces (gray, dashed line). Convergence is slower compared to learning on SFA outputs.
