# Supplementary material for: Reinforcement Learning on Slow Features of High-Dimensional Input Streams
Source: PLoS Comput Biol. 2010 Aug 19;6(8):e1000894. doi: 10.1371/journal.pcbi.1000894 (PMC2924248; doi:10.1371/journal.pcbi.1000894)
Supplement: Figure S2 — Rewards and escape latencies during training of a feed-forward network of simple neurons on the control task with target and distractor. A) Evolution of reward during training. A simulation step for all 100 parallel traces corresponds to 100 time-steps at the x-axis. The plotted values are averages over consecutive 150,000 time steps. B) Evolution of escape latencies (measured in time steps) during training. The number of episodes on the x-axis is the number of completed traces. The plotted values are averages over 8,000 consecutive episodes. (0.02 MB PDF) [file pcbi.1000894.s002.pdf]

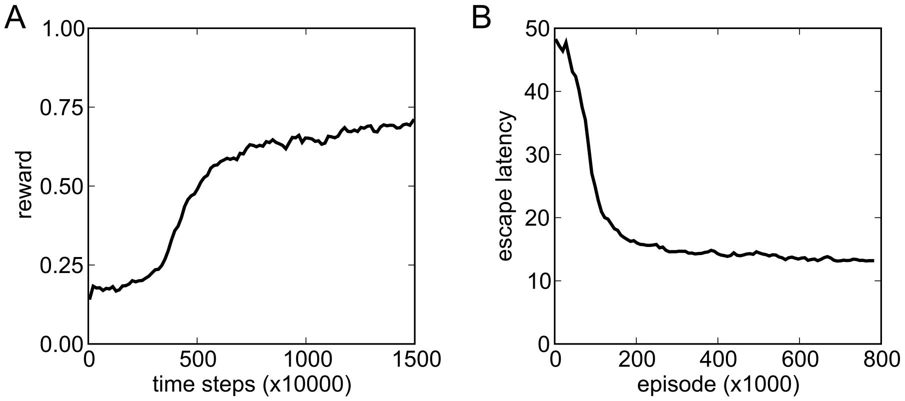

Figure S2: **Rewards and escape latencies during training of a feed-forward network of simple neurons on the control task with target and distractor.** A) Evolution of reward during training. A simulation step for all 100 parallel traces corresponds to 100 time-steps at the x-axis. The plotted values are averages over consecutive 150,000 time steps. B) Evolution of escape latencies (measured in time steps) during training. The number of episodes on the x-axis is the number of completed traces. The plotted values are averages over 8,000 consecutive episodes.
