# Supplementary material for: Reinforcement Learning on Slow Features of High-Dimensional Input Streams
Source: PLoS Comput Biol. 2010 Aug 19;6(8):e1000894. doi: 10.1371/journal.pcbi.1000894 (PMC2924248; doi:10.1371/journal.pcbi.1000894)
Supplement: Text S1 — Detailed parameters for reward-based learning. (0.02 MB PDF) [file pcbi.1000894.s003.pdf]

Supporting Text S1 for Reinforcement Learning on Slow Features of High-Dimensional Input Streams

Robert Legenstein[1], Niko Wilbert[2], and Laurenz Wiskott[2,3]
[1] Institute for Theoretical Computer Science, Graz University of Technology, Austria
[2] Institute for Theoretical Biology, Humboldt-Universität zu Berlin, Germany
[3] Institut für Neuroinformatik, Ruhr-Universität Bochum, Germany

S1: Detailed Parameters for Reward-based Learning

Q-Learning

The weights were initialized as follows. First, all weights were randomly drawn from the uniform distribution in $[0, 1]$. Then the weights for each neuron were normalized to an L2 norm of 0.2. Further parameters were set as follows: $\alpha = 0.8$, $\epsilon = 0.33$, $\eta = 0.02$, $\gamma = 0.95$, and $\sigma = 15$. We have chosen a decaying learning rate $\eta(t) = 0.02 \cdot 0.9995^t$.

Policy-Gradient Learning for neurons with branches

All weights and branch coupling strengths were first initialized to random values uniformly distributed in $[-0.5, 0.5]$. The weight vector $\mathbf{w}_{ik} = (w_{ik0}, \ldots, w_{ikn_{ik}})^T$ of each branch b_{ik} was then normalized to an L2 norm of 0.5 for initialization, where n_{ik} denotes the number of synapses to branch ik. The vectors of branch coupling strengths $\mathbf{u}_i = (u_{i0}, \ldots u_{iK})^T$ were normalized to an L2 norm of 0.1 for initialization. The number of branches per neuron was 100 in all simulations. The noise term ξ was drawn from the uniform distribution over the interval $[-0.5, 0.5]$.

Learning rates were set to $\eta_w = 0.05$ and $\eta_u = 0.001$. We used $\bar{R}(t) = 0.8\bar{R}(t-1) + 0.2R(t)$ for the filtered reward.

Policy-Gradient Learning for two-layer networks of neurons without branches

All weights were first initialized to random values uniformly distributed in $[-0.5, 0.5]$. The weight vector of each neuron in the first layer was then normalized to an L2 norm of 0.5 for initialization. The weight vector of each neuron in the second layer was normalized to an L2 norm of 0.1 for initialization. This initialization procedure is equivalent to the initialization of branch weight vectors and branch strengths in the simulations where neurons with branches were used. The noise term ξ was drawn from the uniform distribution over the interval $[-0.25, 0.25]$. Learning rates of all neurons were set to $\eta = 0.0005$. We used $\bar{R}(t) = 0.8\bar{R}(t-1) + 0.2R(t)$ for the filtered reward.

The choice of parameters was based on simulations with precise state encoding instead of SFA, because simulations with the SFA network were much slower. A learning rate of $\eta = 0.00025$ led to slower convergence, while the network did not converge with a learning rate of $\eta = 0.001$. Noise drawn uniformly over the interval $[-0.5, 0.5]$ caused convergence problems with a learning rate of 0.0005. After the parameters were determined based on the system with precise state encoding, the system was trained with these parameters and the SFA network for preprocessing.
