# Supplementary material for: Reinforcement Learning on Slow Features of High-Dimensional Input Streams
Source: PLoS Comput Biol. 2010 Aug 19;6(8):e1000894. doi: 10.1371/journal.pcbi.1000894 (PMC2924248; doi:10.1371/journal.pcbi.1000894)
Supplement: Text S2 — Derivation of the policy-gradient update rule. (0.02 MB PDF) [file pcbi.1000894.s004.pdf]

Supporting Text S2 for Reinforcement Learning on Slow Features of High-Dimensional Input Streams

Robert Legenstein[1], Niko Wilbert[2], and Laurenz Wiskott[2,3]
[1] Institute for Theoretical Computer Science, Graz University of Technology, Austria
[2] Institute for Theoretical Biology, Humboldt-Universität zu Berlin, Germany
[3] Institut für Neuroinformatik, Ruhr-Universität Bochum, Germany

S2: Derivation of the Policy-Gradient Update Rule

In the following we provide a derivation which shows that the policy-gradient update rules (9) and (10) given in the main text perform gradient ascent on the reward signal $R(t)$. Consider several neurons that influence the reward signal by their output. In fact, in our setup the reward $R(t)$ directly depends on the output of the neurons at time t. The weights should change in the direction of the gradient of the reward signal, which is given by the chain rule as

$$\frac{\partial R(t)}{\partial u_{ik}} = \frac{\partial R(t)}{\partial a_i(t)} \frac{\partial a_i(t)}{\partial u_{ik}} = \frac{\partial R(t)}{\partial a_i(t)} b_{ik}(t), \tag{1}$$

where $a_i(t)$ is the total somatic input to neuron i at time t, see equation (6) in the main text. We assume that the noise ξ is independently drawn at each time and for every neuron with zero mean and variance μ^2, hence we have $\langle \xi_i(t) \rangle = 0$, and $\langle \xi_i(t)\xi_j(t') \rangle = \mu^2 \delta_{ij} \delta(t-t')$ where δ_{ij} denotes the Kronecker Delta, $\delta(t-t')$ denotes the Dirac delta, and $\langle \cdot \rangle$ denotes the average over the random variable ξ, i.e., an average over trials with the same input but different noise. We assume that the noise depends deterministically on the activation of a set of neurons. The deviation of the reward $R(t)$ from the reward $R_0(t)$ without the noise term can be approximated to be linear in the noise for small noise

$$R(t) - R_0(t) \approx \sum_k \frac{\partial R(t)}{\partial a_k(t)} \xi_k(t). \tag{2}$$

Multiplying this equation with $\xi_i(t)$ and averaging over different realizations of the noise, we obtain the correlation between the reward at time t and the noise signal at neuron i

$$\langle (R(t) - R_0(t))\xi_i(t) \rangle \approx \sum_k \frac{\partial R(t)}{\partial a_k(t)} \langle \xi_k(t)\xi_i(t) \rangle = \mu^2 \frac{\partial R(t)}{\partial a_i(t)}. \tag{3}$$

The last equality follows from the assumption that the noise signal is temporally and spatially uncorrelated. Hence, the derivative of the reward signal with respect to the activation of neuron i is

$$\frac{\partial R(t)}{\partial a_i(t)} \approx \frac{1}{\mu^2} \langle (R(t) - R_0(t))\xi_i(t) \rangle. \tag{4}$$

Note also that $\langle R_0(t)\xi_i(t) \rangle = 0$, thus the actual choice of the baseline R_0 is not important in the mean. However, the baseline can have an effect on the variance of the estimate. The choice in the learning rule estimates the average reward for zero noise since inputs are temporally correlated whereas the noise is not. Using this result in equation (1), we obtain

$$\frac{\partial R(t)}{\partial u_{ik}} \approx \frac{1}{\mu^2} \langle (R(t) - R_0(t))\xi_i(t) \rangle b_{ik}(t). \tag{5}$$

We further note that for a small learning rate or if the input changes slowly compared to the noise signal, the weight vector is self-averaging and we can neglect the average in equation (5).

By symmetry, we find that

$$\frac{\partial R(t)}{\partial b_{ik}} \approx \frac{1}{\mu^2} \langle (R(t) - R_0(t))\xi_i(t) \rangle u_{ik}. \tag{6}$$

We thus obtain

$$\begin{aligned} \frac{\partial R}{\partial w_{ijk}} &= \frac{\partial R}{\partial b_{ik}} \frac{\partial b_{ik}}{\partial w_{ijk}} \approx \frac{\partial b_{ik}}{\partial w_{ijk}} \frac{1}{\mu^2} \langle (R(t) - R_0(t))\xi_i(t) \rangle u_{ik} \\ &= \frac{1}{\mu^2} u_{ik} \langle (R(t) - R_0(t))\xi_i(t) \rangle \dot{f}_{ik}(t) x_j(t). \end{aligned}$$
